# Knowledge and Attitude towards Human Papilloma Virus Infection, Vaccines, and Cervical Cancer Prevention among School Students in Kano, Nigeria

**DOI:** 10.1155/2023/2803420

**Published:** 2023-01-04

**Authors:** Ismail Rabiu, Zainab Yahuza

**Affiliations:** Department of Microbiology, School of Science and Information Technology, Skyline University Nigeria, Kano, Nigeria

## Abstract

The rising cases of human papillomavirus (HPV) infection and cervical cancer cases in Nigeria are alarming. Only a few studies have looked at secondary school students in Nigeria's understanding of HPV infection and vaccine acceptance, whereas earlier studies have mostly focused on screening. In this study, 400 students from two secondary schools in Kano State, Nigeria, were engaged with the aim of assessing their level of knowledge and attitudes regarding HPV infection. The study further seeks to understand the respondent's opinion on HPV vaccination and sensitize them to the health effects of HPV infection, thereby communicating the findings to the authorities concerned with policy making. The study revealed that only 128 (32%) and 142 (35.5%) respondents have knowledge about HPV and cervical cancer, respectively. Furthermore, none of the respondents were administered the HPV vaccine, with 81% of them not ready to take the vaccine. It was observed that the majority of the respondents (91%) believed that early hospital visits could help in mitigating HPV or cervical cancer cases. Following their sensitization, the respondents were observed to have different levels of satisfaction, ranging from very satisfied and satisfied to not satisfied. Effective awareness creation amongst students as well as parents is therefore essential in HPV vaccination projects, as well as in reducing the burden of cervical cancer in Nigeria.

## 1. Introduction

Globally, human papillomavirus (HPV) infection is one of the most prevalent sexually transmitted infections. A lifetime exposure to at least one HPV type occurs in more than 50% of sexually active females. Women of all ages can contract HPV, but those between the ages of 20 and 24 have the highest incidence of infection [1, 2]. Almost all sexually active men and women will obtain at least one type of HPV at some point in their lives. Each year, a very high number of new HPV infections in both men and women are reported in Africa. In men, some certain strains of HPV have been found to cause different types of cancer such as cancer of the anus, penis, throat, and mouth [3]. The majority of genital HPV infections are asymptomatic and transitory, and they disappear on their own without producing any disease [4]. Based on the possible risk attached to the disease's progression to malignancy, as well as warts developed in the genital area, these HPV types are categorized as high- or low-risk HPVs. High-risk HPVs consist of HPV16 and HPV18, while HPV types 6 and 11 are low and are mostly associated with genital warts. Cervical cancer, especially the one caused by HPV types (16 and 18), constitutes up to about 70% of cases of all cervical cancers [5].

HPV was found to have a high instance among developing and under-developing countries mainly due to the weak health care system. This might be in connection with the high rate of maternal and child mortality. Over 90% of cases of cervical cancer occur in Africa, with an estimated 569,847 new cases and 311,365 deaths each year [6]. HPV vaccines have the potential to prevent nearly 70% of invasive cervical cancer in women worldwide [7]. Women who have not received the HPV vaccine are at risk of contracting the virus. Given that HPV is the greatest prevalent STD, it is critical that all women are informed about the advantages of vaccination and the hazards associated with not receiving it. Inadequate instruction and understanding regarding the dangers of HPV and the consequences of not getting immunized could result in permanent health difficulties from HPV [1, 8].

Studies have indicated that in various African nations, the significance of understanding HPV infection and the level of public acceptability of vaccinations vary from low to high. In Africa, 8–17 kids are heavily involved in vaccination programs, which primarily work to avoid infantile illnesses [9]. Therefore, a program to prevent cervical cancer will be more successful if students are aware of the infection, have an understanding of it, and develop a positive attitude toward HPV vaccination. In Nigeria's understanding of HPV infections, including vaccine acceptability, only a few studies have looked at secondary school students, whereas earlier research has mostly focused on screening. However, one of the most crucial steps for the prevention and treatment of cervical cancer is increasing knowledge and persuading individuals to have a favorable attitude toward the HPV vaccination [1]. This study aims to access the knowledge and attitude toward HPV infection, their vaccines, and cervical cancer prevention among secondary school students in Kano, Nigeria.

## 2. Materials and Methods

### 2.1. Study Site

Two secondary schools in Kano (Professor Hafsat Abdullahi Ganduje Secondary School Gyadi-Gyadi in Tarauni Local Government and Government Girls Secondary School Gandu in Kano Municipal Local Government, Kano State, Nigeria, were engaged in this study. Kano state shares the GPS coordinates of 11.7471° N and 8.5247° E. The state shares a boundary with Bauchi, Katsina, Jigawa, and Kaduna states on the Southeast, Northwest, Northeast, and Southwest, respectively [10].

### 2.2. Study Design

**Figure 1:**
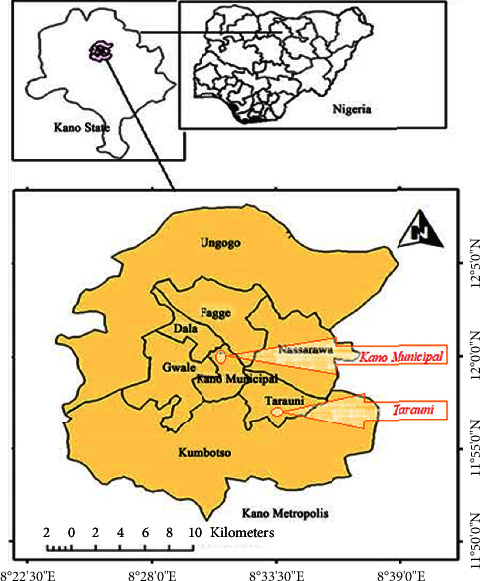
Map of Kano State showing the sampling site “Kano Municipal City and Tarauni Local Government” and states with which Kano shares boundary with (10).

A cross-sectional study focused on two schools was carried out in Kano (Figure 1), Nigeria, between the 5^th^ of March and the 27^th^ of August, 2022.

### 2.3. Sampling Technique

A random sampling technique was employed among each respondent that meets the inclusion criteria.

### 2.4. Study Population

Secondary students aged 10 and above.

### 2.5. Sample Size

Using the formula (*n*)=*Z*1 − *α*^2^/2*P*(1 − *P*)/*d*^2^ and a prevalence of 11.1% as reported by Chigozie et al. [2], in a study conducted among female students at the University of Lagos, Lagos, Nigeria, a sample size of (*n*) = 150 was calculated. The sample size was approximated to be 200 for each of the two schools, to give a total sample size of 400 in all two schools.

### 2.6. Inclusion Criteria

Inclusion criteria were as follows:

- Students whose consent was sought and agreed to participate
- Girls aged 10 and above

### 2.7. Exclusion Criteria

Exclusion criteria were as follows:

- Students whose consent was sought and agreed not to participate
- Girls below 10 years

### 2.8. Ethical Approval

Ethical approval was obtained from each of the schools before the commencement of the study, with the agreement to communicate the findings of the study to the school's management for effective policy making. Consent of each participant was sought for prior to their engagement in the study.

### 2.9. Statistical Analysis

Data obtained were analyzed using SPSS version 25 and presented in percentages.

## 3. Results and Discussion

### 3.1. Results

The respondents from the two schools (Professor Hafsat Abdullahi Ganduje (PHAG) Secondary School Gyadi-Gyadi and Government Girls Secondary School (GGSS) Gandu) were accessed on the level of knowledge and attitude towards HPV and vaccine acceptance (Table 1). In this regard, respondents that choose “No” were observed to have the highest response.

The respondents were assessed on the risk factors (Table 2) that may be linked to HPV among the respondents. They were observed to stand a very low risk of contracting HPV infection as many risks of HPV including signs were found not to be associated with the majority of the respondents.

The demographic characteristics of the respondents (Table 3) shows that the majority of the respondents are within the age category of 15–19 years of age, 99% did not marry, and with 46% were engaged in trading during holidays.

Key points that relate to HPV and cervical cancer were used to sensitize students to, as well as the practice of healthy lifestyles in order to prevent themselves from contracting deadly HPV infections. This was followed by (Table 4) by their feedback on their level of satisfaction. The students were observed to have much level of satisfaction following the conclusion of the sensitization.

### 3.2. Discussion of Results

This study shows that the majority of the respondents do not have basic knowledge about both HPV infections and cervical cancer (Table 1), with only 32% and 142 (35.5%) of the respondents having knowledge about HPV and cervical cancer, respectively. None of the respondents were administered the HPV vaccine, while 81% of the respondents are not ready to be administered the HPV vaccine. Furthermore, the majority (91%) of the respondents believed that early hospital visits could help in mitigating HPV or cervical cancer cases. HPV is one of the most widespread sexually transmitted diseases in the world [4, 11]. People's negative perception of the vaccine as well as the means of disease spread might be the guiding factor behind the vaccine rejection. Furthermore, the less level of awareness among healthcare providers might be the reason there is less level of awareness [12–14].

This study also finds out that none of the respondents have conducted any laboratory tests on HPV infection or cervical cancer. Only 41% (166) and 13% (53) (Table 2) of the respondents had some history of the presence of genital warts/skin lesions or any pain in the genital area and a history of experiencing any form of vaginal discharge respectively. On the history of experiencing any infection associated with the genital area or urinary tract and unusual bleeding in the genital area, only 91 (23%) and 14 (4%) were recorded, respectively. On the history of experiencing frequent stomach pain, the family history of any illness associated with excessive genital bleeding, and the family history of any member ever been diagnosed with cancer, only 179 (45%), 71 (17%), and 23 (6%) of the respondents were found to have a level of connection with it. This made the respondents to stand a lower risk of contracting HPV infection, as most of the early signs and symptoms of HPV infection were found not to be associated with the respondents, respectively. The knowledge of the risk factors of HPV was found to be higher in countries that have existing HPV control strategies, which include sensitizations in rural communities, hospitals, as well as among school students [8, 15, 16].

The result further shows that the majority of the respondents 265 (66%) were within the 15–19 years category, with none falling in the 25 years and above category (Table 3). Regarding marital status, 396 (99%) of the respondents are single, with only 1% of them being married. The majority (46%) of the respondents engaged in trading during their holidays, with very few (22%) of them not engaged in any occupation during the period of their holidays. PHAG Gyadi-Gyadi had the least number of respondents 3 (1.5%) with a history of pregnancy, while GGSS Gandu had 7 (3.5%). However, based on the number of children of the respondents, 398 (99.5%) had no children, with only 2 (0.5%) having 1–3 children. Considering the high number of respondents that are not married and the less number of children among the very few that are married, these might be in connection with the less or absence of any knowledge about the existence of HPV and cervical cancer, considering the increasing level of awareness created among expectant or nursing mothers during their visit to hospitals. Different research studies have stressed the importance of the spread and detection of HPV in children, infants, and women. Furthermore, several research findings have demonstrated that HPV can be spread to infants from their mothers, especially during childbirth, with high chances of infection with the high-risk HPV strains [17–19].

**Figure 2:**
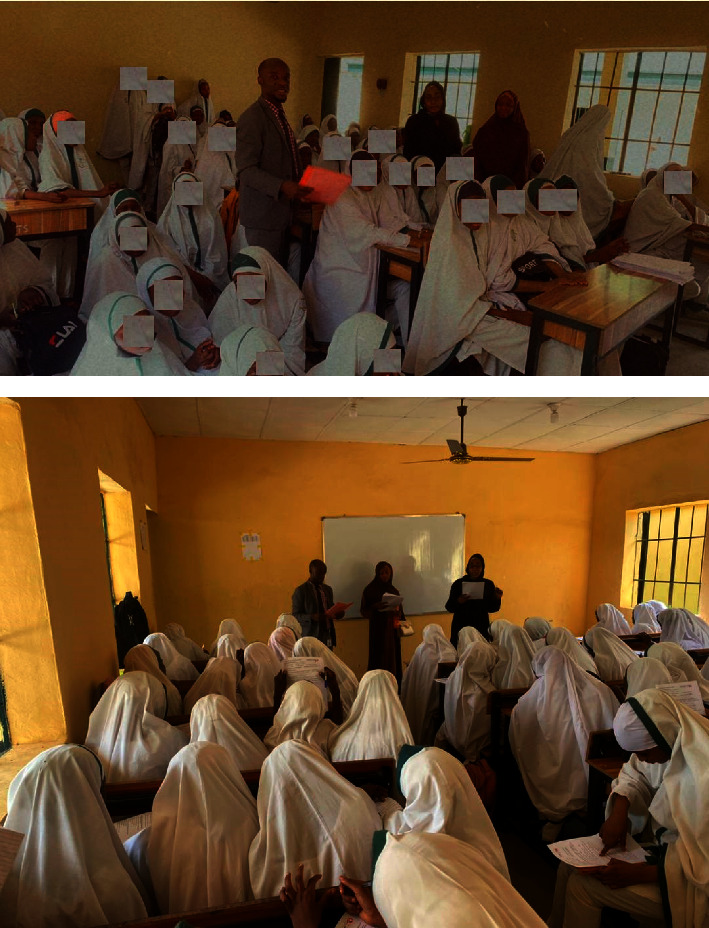
Sensitization on the health effect of HPV and cervical cancer conducted at Professor Hafsat Abdullahi Ganduje Secondary School Gyadi-Gyadi, Kano State, Nigeria.

**Figure 3:**
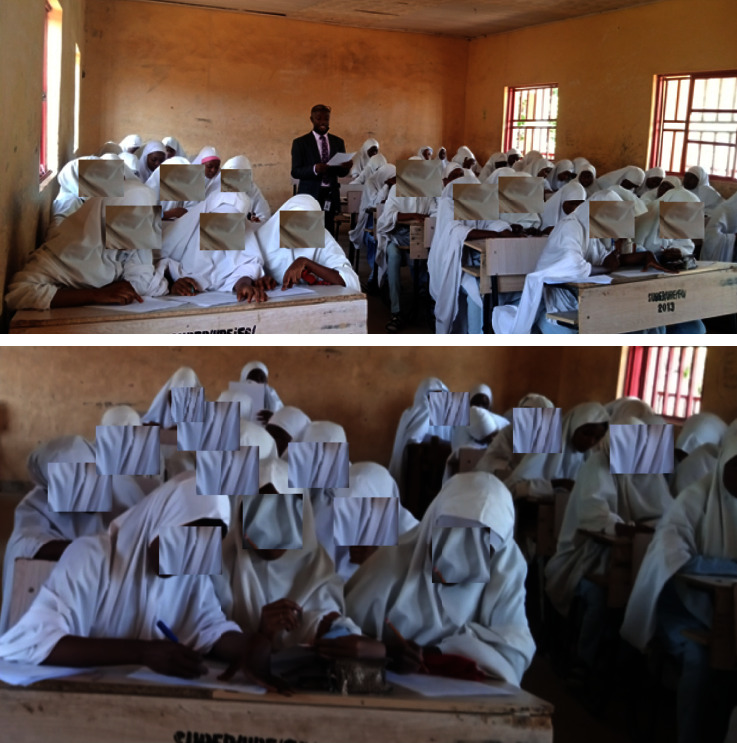
Sensitization on the health effect of HPV and cervical cancer conducted at Government Girls Secondary School Gandu, Kano State, Nigeria.

Following the conclusion of the sensitization (Figures 2 and 3) and the collation of feedback, the respondents were observed to have different levels of satisfaction ranging from very satisfied and satisfied to not satisfied. In the two schools, PHAG Gyadi-Gyadi was observed to have a higher level of respondents who are satisfied with the level of awareness created (Table 4). This might be due to the nature of the two schools: PHAG Gyadi-Gyadi is a science-based school, whereas Government Girls Secondary School Gandu is mixed, having students of Science, Arts, and Management backgrounds. Sensitization questions that got the highest level of satisfaction are “Can women get HPV (97%), What is HPV infection (91%), Is HPV curable (91%), Does HPV cause cancer (86%), what is the risk of cervical cancer after HPV infection (89%), and Can HPV be transmitted from the mother to the baby during pregnancy (86%);” this might be in a connection that the questions are not tricky and difficult to comprehend by the respondents. Furthermore, the level of satisfaction obtained among the respondents is actually encouraging, especially for the fact that this is the first time sensitization on cancer was carried out in the two schools. In Table 1, research question (iv) explained the number of participants that are willing to take the vaccine which was very less, as the majority of the respondents were of the opinion that they will not take the vaccine possibly as a result of the unsure of its safety and effectiveness. Before the sensitization, respondents ready to take the HPV vaccine was only 49 and 27 from PHAG and GGSS, respectively. Whereas after the sensitization, there is an increase in the number of vaccine acceptance, as well as willingness to take the vaccine increases (Table 4 research question (xv)). This was possibly as a result of the sensitization which makes them well-informed of the importance of the vaccine in the prevention of HPV infections. Moreover, conducting public talks, discussions, and\or awareness campaigns on diseases that has to do with the genital area is actually something very new and unwelcoming especially in Kano State, a core Muslim state located in the northern part of Nigeria with the Hausa tribe as the majority. There is no doubt that this plays a key role in keeping the majority of the populace ill-informed, especially those from rural areas on various means of protection, prevention, and disease treatment. Furthermore, the traditional view of limiting HPV and cervical cancer disease transmission to only via sexual intercourse needs to be addressed as the literature survey reveals many different routes of disease transmission, especially blood transfusion, sharp objects, along with other contact with blood or bodily fluids [6, 20, 21]. The findings of this study were as well communicated with the school authorities and the Kano State Ministry of Health for effective policy making.

## 4. Conclusions

This research work employed a total of 400 students attending Professor Hafsat Abdullahi Ganduje Secondary School Gyadi-Gyadi and Government Girls Secondary School Gandu, Kano State, Nigeria. The study revealed that female students at the secondary school of Kano often had little understanding of or perception of their susceptibility to HPV infection and disorders connected to HPV. Furthermore, the majority of the respondents are ignorant of HPV, cervical cancer, and various means of disease transmission. Furthermore, most of the respondents are not ready to be administered with vaccines. Considering the high level of satisfaction among the respondents following sensitization on some important means of disease spread, control, and the urgent need to see a doctor whenever some signs associated with risk factors are seen, one cannot overstate the need for developing an effective HPV education program to close the knowledge gap.

## 5. Recommendation

- There is a need for parents to engage in creating awareness on preventive measures for all sexually transmitted diseases, with HPV inclusive among their female children
- There is a concern among parents that HPV vaccination usually leads to a higher level of sexual activity among adolescents. For this reason, adequate orientation needs to be made among parents.
- Sexual health education should be introduced in secondary schools with a view to educating the populace
- For a successful HPV vaccination project in Nigeria, there is the need to carry out both traditional leaders, religious leaders, local radio, and TV stations to create awareness amongst parents and the general populace with a view to dispel the negative public views regarding HPV vaccination.

## Figures and Tables

**Table 1:** Level of knowledge and attitude towards HPV and vaccine acceptance among the respondents in the study area.

S/No.	Research question	PHAG Gyadi-Gyadi (*n* = 200)		GGSS Gandu (*n* = 200)		Total (*n* = 400)	
		Yes	No	Yes	No	Yes (%)	No (%)
(i)	Knowledge about HPV	86	114	42	158	128 (32)	272 (68)
(ii)	Knowledge about cervical cancer	97	103	45	155	142 (35.5)	258 (64.5)
(iii)	Respondents that have been administered the HPV vaccine	0	200	0	200	0	400 (100)
(iv)	Respondents ready to take the HPV vaccine	49	151	27	173	76 (19)	324 (81)
(v)	Do you believe that early hospital visits help in HPV or cervical cancer cases?	191	9	173	27	364 (91)	36 (9)

PHAG, Professor Hafsat Abdullahi Ganduje Secondary School Gyadi-Gyadi; GGSS, Government Girls Secondary School Gandu.

**Table 2:** Assessment of risk factors that may be linked to HPV among the respondents.

S/No.	Research question	PHAG Gyadi-Gyadi		GGSS Gandu		Total	
		Yes	No	Yes	No	Yes (%)	No (%)
(i)	Respondents that have conducted any laboratory tests on HPV infection or cervical cancer	0	200	0	200	0	400 (100)
(ii)	History of any experience of the presence of genital warts/skin lesions or any pain in the genital area by the respondent	77	123	89	111	166 (41)	234 (59)
(iii)	History of experiencing any form of vaginal discharge	21	179	32	168	53 (13)	347 (87)
(iv)	History of any infection associated with the genital area or urinary tract by the respondent	44	156	47	153	91 (23)	309 (77)
(v)	History of experiencing any unusual bleeding in the genital area by the respondent	3	197	11	189	14 (4)	386 (96)
(vi)	History of experiencing frequent stomach pain	75	125	104	96	179 (45)	221 (55)
(vii)	Family history of any illness associated with excessive genital bleeding	51	149	20	180	71 (17)	329 (82)
(viii)	Family history of any member ever been diagnosed with cancer	14	186	9	191	23 (6)	377 (94)

PHAG, Professor Hafsat Abdullahi Ganduje Secondary School Gyadi-Gyadi; GGSS, Government Girls Secondary School Gandu.

**Table 3:** Demographic characteristics that may be linked to the occurrence of HPV among the respondents.

S/No.	Characteristics		PHAG Gyadi-Gyadi (*n* = 200) (%)	GGSS Gandu (*n* = 200) (%)	Total (*n* = 400) (%)
(i)	Age (years)	10–14	46	33	79 (20)
		15–19	127	138	265 (66)
		20–24	27	29	56 (14)
		≥25	0	0	0

(ii)	Marital status	Single	199	197	396 (99)
		Married	1	3	4 (1)
		Divorce	0	0	0

(iii)	Occupation during holidays	Trading	88	95	183 (46)
		Shop keeping	72	57	129 (32)
		None	40	48	88 (22)

(iv)	History of pregnancy	Yes	3 (1.5)	7 (3.5)	10 (2.5)
		No	197 (98.5)	193 (96.5)	390 (97.5)

(v)	Number of children	None	200	198	398 (99.5)
		1–3 children	0	2	2 (0.5)
		≥4	0	0	0

PHAG, Professor Hafsat Abdullahi Ganduje Secondary School Gyadi-Gyadi; GGSS, Government Girls Secondary School Gandu.

**Table 4:** Questions on which the respondents were sensitized along with their feedback following their sensitization.

S/No.	Sensitization questions	Level of satisfaction									
		PHAG Gyadi-Gyadi (*n* = 200)			GGSS Gandu (*n* = 200)			Total (*n* = 400)		
		A	B	C	A	B	C	A (%)	B (%)	C (%)
(i)	What is HPV infection?	193	7	0	173	12	15	366 (91.5)	19 (4.75)	15 (3.75)
(ii)	What are the symptoms of an HPV infection?	189	11	0	155	28	17	344 (86)	39 (9.75)	17 (4.25)
(iii)	What are HPV genital warts?	133	53	14	146	23	31	279 (69.75)	76 (19)	45 (11.25)
(iv)	How do we contract HPV?	158	41	1	178	11	11	336 (84)	52 (13)	12 (3)
(v)	How serious is HPV?	167	29	4	177	23	0	344 (86)	52 (13)	4 (1)
(vi)	Is HPV curable?	195	5	0	169	24	7	364 (91)	29 (7.25)	7 (1.75)
(vii)	Which treatment would you recommend for HPV infection?	143	49	8	159	40	1	302 (76)	89 (22.25)	9 (2.25)
(viii)	Can I get tested for HPV?	189	11	0	144	0	56	333 (83)	11 (2.75)	56 (14)
(ix)	Does HPV cause cancer?	184	4	12	158	22	20	342 (86)	26 (6.5)	32 (8)
(x)	Can women get HPV?	198	0	2	188	2	10	386 (97)	2 (0.5)	12 (3)
(xi)	Can I acquire HPV infection from my husband?	150	39	11	165	31	4	315 (79)	70 (17.5)	15 (3.75)
(xii)	How long does it take for HPV infection to show in my body?	197	0	3	134	60	6	331 (83)	60 (15)	9 (2.25)
(xiii)	I find a small mass on genital area, is it a genital wart?	143	51	6	151	0	49	294 (74)	51 (12.75)	55 (13.75)
(xiv)	What is the risk of cervical cancer after HPV infection?	198	2	0	156	44	0	354 (89)	46 (11.5)	0
(xv)	How effective is the HPV vaccination?	133	41	26	89	46	65	222 (56)	87 (21.75)	91 (22.75)
(xvi)	If I am infected with HPV, and my husband has other wives, will they also be infected?	162	38	8	148	36	8	310 (78)	74 (18.5)	16 (4)
(xvii)	Is vaginal discharge related to the symptoms of HPV infection?	177	23	0	122	23	55	299 (75)	46 (11.5)	55 (13.75)
(xviii)	Can HPV be transmitted from the mother to the baby during pregnancy?	166	32	2	179	12	9	345 (86)	44 (11)	11 (2.75)
(xix)	Is there anything I can do to prevent HPV infection?	196	4	0	147	5	48	343 (86)	9 (2.25)	48 (12)
(xx)	How can I help my other family members to get protected from HPV infection?	157	42	1	150	37	13	307 (77)	79 (19.75)	14 (3.5)

A, very satisfied; B, satisfied; C, not satisfied; PHAG, Professor Hafsat Abdullahi Ganduje Secondary School Gyadi-Gyadi; GGSS, Government Girls Secondary School Gandu.

## Data Availability

The data used to support the findings of this study are included within the article.
